# The evolution of the Earth’s surface iron cycle

**DOI:** 10.1073/pnas.2608784123

**Published:** 2026-08-05

**Authors:** Florian Scholz, Sebastian Doetterl, Dalton S. Hardisty

**Affiliations:** ^a^ Department of Earth System Sciences, University of Hamburg, Hamburg 20146, Germany; ^b^ Department of Environmental Systems Science, ETH Zürich, Zürich 8092, Switzerland; ^c^ Department of Earth and Environmental Sciences, Michigan State University, East Lansing, MI 48824

**Keywords:** iron cycle, chemical weathering, tectonics, oxygen, evolution

## Abstract

Formation and burial of pyrite (iron sulfide) in marine sediments exert a fundamental control on atmospheric oxygenation and seawater buffering over geological timescales. However, little is known about how the formation and delivery of its precursor, reactive iron (Fe) oxide minerals, have evolved throughout Earth history. Secular variability in reactive Fe (including Fe oxides and pyrite) preserved in marine sediments is commonly attributed solely to redox changes. Here, we develop an approach to distinguish redox-driven influences from other controls on sedimentary reactive Fe, including the intensity of continental silicate weathering and the transfer of terrigenous particles to the ocean. We apply this framework to a compilation of reactive Fe data spanning 1,200 My of Earth history. Our results reveal persistently low proportions of reactive Fe from the Mesoproterozoic through the Cambrian, followed by a pronounced mid- to late Paleozoic rise and a subsequent decline in the late Cenozoic. This temporal pattern is inconsistent with a purely redox-driven control. Comparison with independent proxies for continental weathering and land-ocean sediment and solute fluxes suggests a strong coupling between reactive Fe burial, oxidative silicate weathering, and tectonically driven erosion. Notably, the mid- to late Paleozoic rise in reactive Fe coincided with the proliferation of land plants and increasing atmospheric oxygen. We propose a positive feedback where Earth-surface Fe cycling was both amplified by atmospheric oxygenation and contributed to it through its influence on pyrite burial in marine sediments.

## Significance

Iron oxide minerals produced on land by chemical weathering are transported to the ocean and buried in marine sediments, where they can form pyrite or bind to organic matter, processes that release oxygen and influence atmospheric oxygenation over geological timescales. Here, we show that the production and delivery of iron oxides to the ocean have varied over the past 1,200 My, driven by changes in weathering intensity controlled by land plant evolution and plate tectonics. These findings reveal a critical link between Earth’s surface processes and the long-term oxygenation of the atmosphere.

Iron (Fe) is a key element of the Earth system, coupling the biogeochemical cycles of carbon, oxygen, sulfur, and numerous other bioactive elements. Because Fe serves as an essential cofactor in carbon and nitrogen fixation, the supply of bioavailable Fe limits a substantial fraction of global marine primary production (1). Iron oxide minerals deposited on the seafloor may undergo reductive dissolution, releasing dissolved Fe into pore waters (2). Under low-oxygen conditions in bottom waters, a portion of this dissolved Fe can be transferred across the sediment–water interface, supporting primary production in the overlying ocean (3, 4). An important fraction of Fe oxides delivered to the seafloor reacts with hydrogen sulfide produced from microbial sulfate reduction to form pyrite (FeS_2_) (5, 6). Burial of pyrite in marine sediments results in a net accumulation of alkalinity (7, 8) and oxygen (9, 10) in Earth’s surface reservoirs, influencing the buffering capacity of seawater and the long-term trajectory of Earth’s oxygenation. Sedimentary Fe oxide minerals that escape reductive dissolution may associate with organic matter, protecting it from degradation and driving its removal from surface reservoirs through long-term burial (11). This Fe burial pathway further contributes to the accumulation of atmospheric oxygen over geological timescales (12).

Despite the key role of Fe in shaping carbon sequestration, sulfur burial, and Earth’s oxygenation, relatively little is known about how the delivery of reactive iron to marine sediments has varied throughout Earth’s history. Reactive Fe refers to the sum of Fe species that can react with hydrogen sulfide to form pyrite on timescales relevant to Earth-surface processes (6, 13), primarily including ferric Fe hydroxides and oxides such as ferrihydrite (Fe(OH)_3_), goethite (FeOOH) and hematite (Fe_2_O_3_). A variety of wet chemical extraction techniques are routinely employed to quantify the operationally defined highly reactive Fe pool (Fe_HR_) in marine sediments (14, 15). The sum of oxidized and reduced (i.e., pyrite) reactive Fe species is widely used as a paleo-redox indicator, where a high fraction of reactive Fe relative to total Fe (Fe_HR_/Fe_T_) indicates anoxic conditions at the time of deposition (13). Reactive Fe enrichments relative to the terrigenous background are thought to result from the mobility of iron under anoxic conditions, including reductive dissolution of Fe oxides, transport, and reprecipitation (16). Under this latter scenario, sediments are also expected to display elevated ratios of total Fe to aluminum (Al), because Fe is redox-sensitive whereas Al is not (17, 18). As an additional Fe speciation proxy, the fraction of pyrite in the highly reactive Fe pool (Fe_PY_/Fe_HR_) is used to evaluate the availability of hydrogen sulfide in paleo-environments (13). Importantly, reconstructions of Fe_HR_/Fe_T_ and Fe_PY_/Fe_HR_ have served as foundational proxies for constraining the extent of anoxia and the accumulation of dissolved Fe or hydrogen sulfide in ancient oceans, conditions referred to as ferruginous and euxinic, respectively (19–22).

Paleo-biogeochemical studies commonly apply a single threshold range in Fe_HR_/Fe_T_ to identify anoxic conditions (13), regardless of sample location or geological age. However, this approach overlooks the influence of factors beyond redox conditions in bottom waters and surface sediments on sedimentary Fe speciation. Iron oxide minerals are produced on land through the chemical weathering of Fe-bearing silicate minerals (e.g., pyroxene, olivine, and biotite) (23, 24). Depending on climate variables such as temperature and precipitation, as well as tectonically driven erosion rates, material transported through river catchments may be variably enriched in reactive Fe (25, 26). In dynamic sedimentary environments of estuaries and deltas, catchment-derived reactive Fe signals may be modified by reverse weathering processes during which authigenic silicate minerals (e.g., glauconite, Fe-rich smectite) are formed from terrestrial Fe and Al oxides, biogenic silica, and cations dissolved in seawater (27–29). Consequently, the record of reactive Fe in marine sediments could preserve information about the intensity of chemical weathering (and reverse weathering) through geologic time. Importantly, these processes are an essential component of the long-term carbon cycle regulating atmospheric carbon dioxide and Earth surface temperature on geological timescales (30–32).

In this study, we use reactive Fe data from modern sediments, soils, and river suspended particles to develop a general framework for disentangling redox- and weathering-related reactive Fe signals in marine sediments. We then apply this framework to a compilation of sedimentary Fe speciation data spanning Earth’s history from the mid-Proterozoic to the present. Our analysis reveals a pronounced increase in reactive Fe starting in the early to middle Paleozoic, coinciding with the evolution and expansion of land plants and the cyclic influence of chemical and physical erosion driven by plate tectonics. These observations suggest a tight coupling between the evolution of the low-temperature Fe cycle and the broader Earth system.

## Results

### Environmental Controls on Reactive Iron in Modern Marine Sediments.

Variability in Fe_HR_/Fe_T_ in ancient marine sediments is commonly attributed to enhanced mobility of dissolved ferrous iron (Fe^2+^) under anoxic conditions (16). When reactive Fe is either lost from or added to the sediment, Fe_HR_/Fe_T_ and Fe/Al ratios decrease or increase, respectively, relative to the background terrigenous input. Fe_HR_/Fe_T_ values > 0.38 are generally considered indicative of deposition under anoxic conditions, whereas ratios between 0.22 and 0.38 are regarded as equivocal (13). These thresholds were derived from a compilation of modern marine sediments that were deposited beneath oxic bottom water, which display an average Fe_HR_/Fe_T_ of 0.28 (33).

**Fig. 1.**
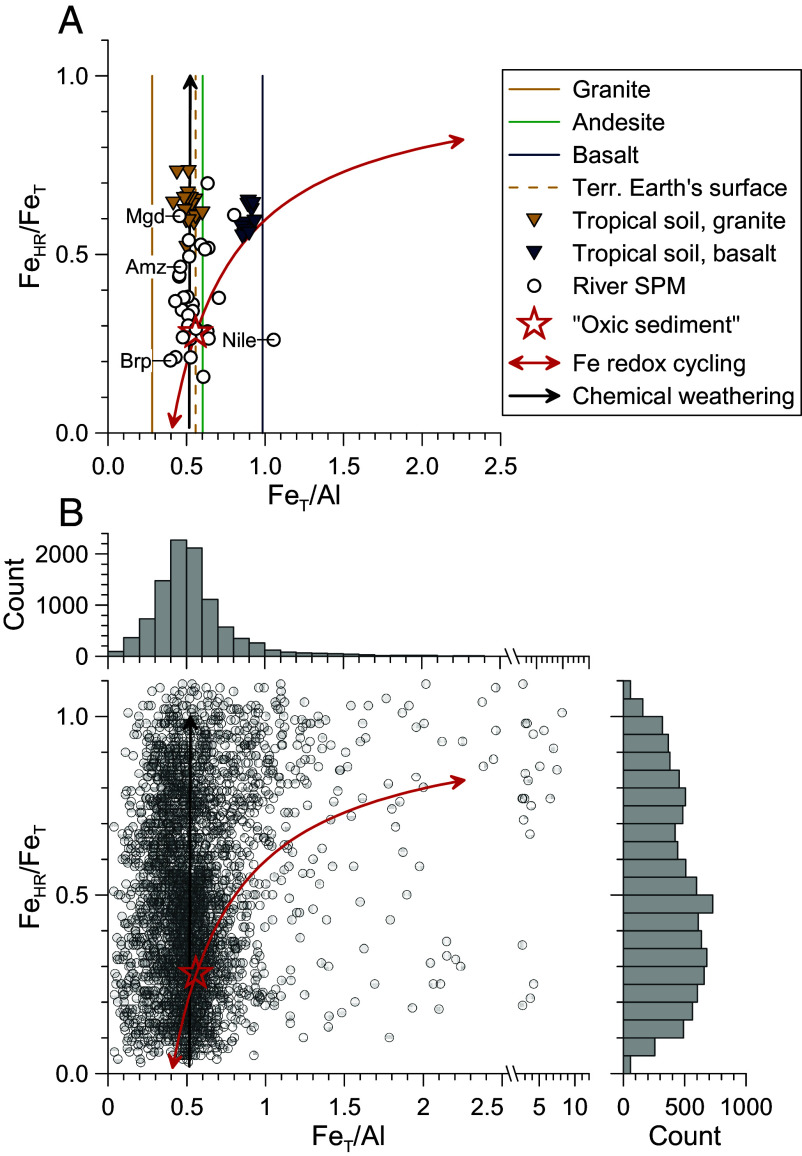
Impact of continental chemical weathering and oceanic redox processes on sedimentary Fe speciation. (*A*) Compilation of Fe speciation data for different rock types (37), the modern terrestrial Earth’s surface (37), tropical soils (this study), and suspended particles (SPM) from 22 rivers in the United States (25), as well as from eight additional rivers worldwide (26), including the Amazon River (Amz), Brahmaputra River (Brp), Magdalena River (Mgd), and Nile River. (*B*) Compilation of sedimentary Fe speciation data from marine sediments spanning the past 1,200 Ma. The red star (“oxic sediment”) represents the Fe_HR_/Fe_T_ ratio of modern marine sediments underlying oxic bottom water as defined by ref. 33, combined with the Fe_T_/Al of the modern terrestrial Earth’s surface (37). The curved trend line depicting marine Fe redox cycling was calculated using Eq. **5**, with the “oxic sediment” composition as the terrigenous baseline (Fe_T,in_ = 1.0, Fe_HR,in_ = 0.28, Al_in_ = 1.79). Adoption of an alternative terrigenous background would shift the position and curvature of the trend line (see *SI Appendix* for examples). The near-vertical trend line illustrates the effect of chemical weathering.

If redox-driven iron mobility is the sole process controlling the composition of Fe revealed by the Fe speciation proxy, Fe_HR_/Fe_T_ and Fe_T_/Al would follow a predictable asymptotic relationship (Fig. 1*A*; see *Methods* for details) (18). This pattern has been documented in modern and ancient marine environments where reactive Fe is reductively mobilized, transported, and subsequently reprecipitated at a different location (18, 34–36). Depending on whether reactive Fe is lost or gained, Fe speciation ratios are expected to shift toward lower or higher values relative to the oxic reference Fe_HR_/Fe_T_ and Fe_T_/Al of the modern Earth’s surface (Fig. 1*A*).

However, other processes within Earth’s low-temperature iron cycle can also fractionate Fe_HR_ relative to Fe_T_ while leaving Fe_T_/Al largely unchanged. During oxidative chemical weathering, relatively unreactive Fe bound in silicate minerals is transformed into iron oxide phases, increasing the proportion of highly reactive iron (23, 24). In contrast, the bulk Fe_T_/Al ratio typically remains similar to that of the unweathered parent rock because both Fe and Al are generally insoluble and remain in the solid phase during weathering under circumneutral to mildly acidic pH conditions. This relationship is illustrated by comparing the average compositions of various rock types with those of tropical soils and river suspended particles in a plot of Fe_HR_/Fe_T_ vs. Fe_T_/Al (Fig. 1*A*). Felsic igneous rocks, such as granite, contain fewer Fe-rich aluminosilicate minerals than mafic igneous rocks such as basalt and therefore exhibit lower Fe_T_/Al ratios. This primary Fe_T_/Al signature is largely preserved during chemical weathering, even under the intense weathering regime of tropical soils.

At the catchment scale, the Fe_HR_/Fe_T_ ratio of river-transported particles has been shown to covary with runoff and the intensity of chemical weathering (25). The degree of chemical weathering is generally governed by temperature, water availability, and the residence time of material in the weathering zone, which is ultimately controlled by tectonic uplift and erosion rates (30). Under conditions where bedrock is exposed to elevated temperatures and humid conditions for prolonged periods, a greater proportion of silicate-bound Fe can be transformed into reactive Fe, resulting in higher Fe_HR_/Fe_T_ ratios in river suspended particles (25). Accordingly, suspended particles from rivers draining low-relief tropical terrains (e.g., Magdalena River and Amazon River) typically exhibit higher Fe_HR_/Fe_T_ ratios than those from mountainous catchments where particles are rapidly eroded (e.g., Brahmaputra River) (Fig. 1*A*). Consistent with the limited influence of weathering on Fe_T_/Al ratios, river suspended particles generally display Fe_T_/Al values similar to the average composition of the terrestrial Earth’s surface. An exception is the Nile River, which drains basaltic terrain in the Ethiopian Highlands, resulting in distinctive Fe/Al ratios that reflect its catchment geology (38).

Taken together, tropical soil samples and river suspended particles define an array of increasing Fe_HR_/Fe_T_ at largely constant Fe_T_/Al with increasing weathering intensity (Fig. 1*A*). Reverse weathering in the land–ocean transition zone could, in principle, decrease the Fe_HR_/Fe_T_ of river suspended particles by converting Fe oxide minerals into authigenic silicates (27–29). However, sediments on the Amazon shelf, a classic setting for reverse weathering, retain their river-derived Fe_HR/_Fe_T_ signatures during transport from the river mouth to the deep sea (28). Moreover, reverse weathering does not alter Fe content relative to Al and therefore does not displace samples from the chemical weathering array in Fig. 1*A*. In the following, we use this array to distinguish weathering-driven trends from redox-related variations in Fe speciation over geological time.

### Sedimentary Reactive Iron Across the Past 1,200 Mya.

Our primary objective is to evaluate Earth’s surface Fe cycling in the context of Neoproterozoic to Paleozoic oxygenation events, major orogenic episodes, and associated biological innovations. To this end, we analyze sedimentary Fe speciation data, alongside independent proxy records of weathering and redox conditions, spanning the past 1,200 My. Our database comprises roughly 10,000 individual data points (Fig. 1*B*), most of which are derived from the comprehensive database of the Sedimentary Geochemistry and Paleoenvironments Project (SGP) (39), supplemented by approximately 1,000 additional samples, mostly representing modern or Quaternary continental margin sediments, as well as samples from the Cenozoic and late Cretaceous (see *Methods* for details).

Most of the samples contained in our database were deposited in continental margin settings or epicontinental basins. Consequently, sedimentary Fe speciation may theoretically reflect the combined influence of continental weathering, marine redox cycling, and postdepositional processes. Postdepositional processes include the pyritization or alteration of poorly reactive Fe phases, such as sheet silicates, through reactions with H_2_S produced during anaerobic methane oxidation coupled to sulfate reduction, or with CO_2_ and methane generated during methanogenesis (40–42). If the products of these reactions are more readily extracted than their precursor phases, they would lead to an increase in Fe_HR_/Fe_T_ while conserving Fe_T_/Al (Fig. 1).

**Fig. 2.**
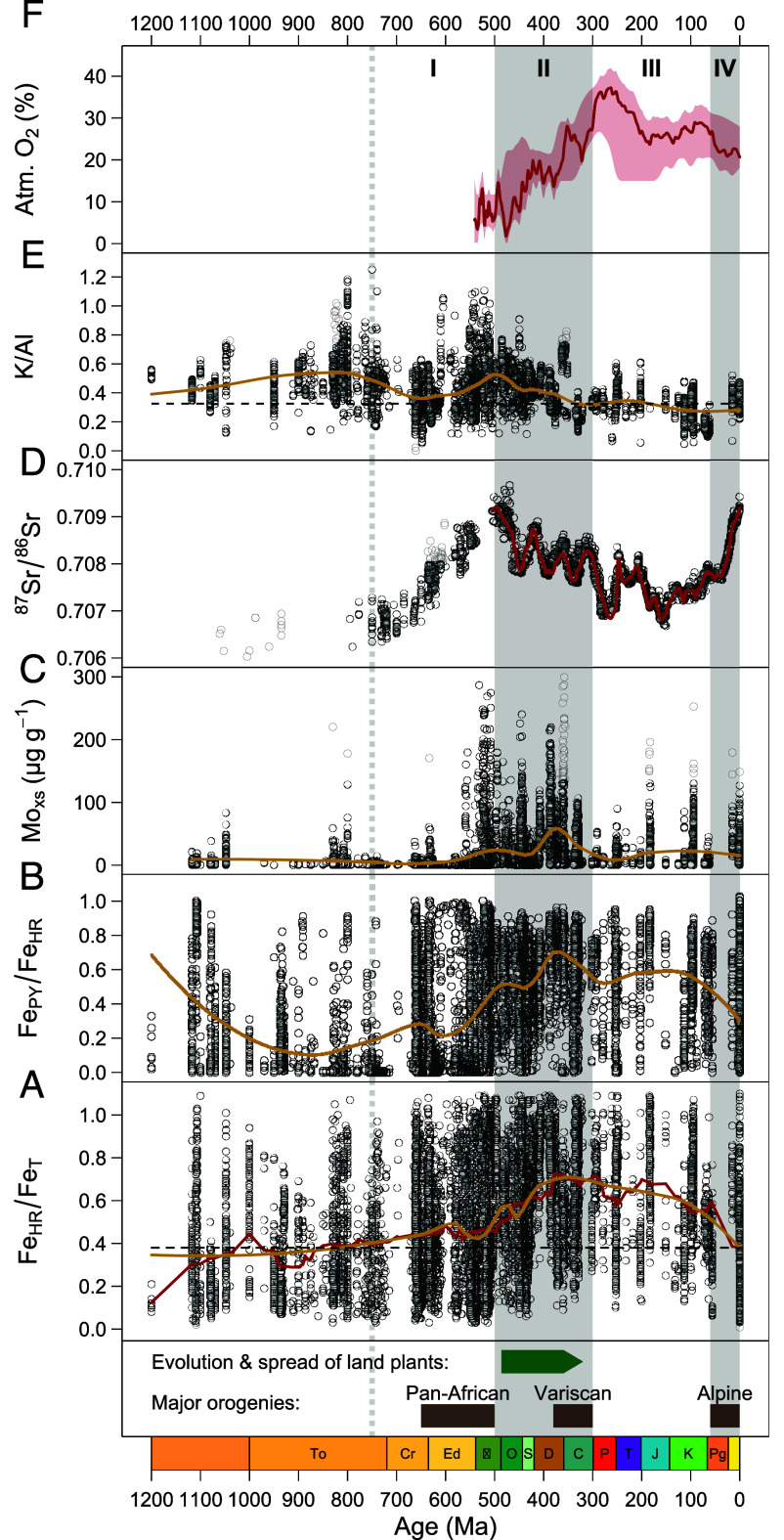
Weathering and redox proxies over the past 1,200 Ma. (*A*) Fe_HR_/Fe_T_; (*B*) Fe_PY_/Fe_HR_; (*C*) Mo_XS_, (*D*)^87^Sr/^86^Sr (43, 44); (*E*) K/Al; (*F*) modeled evolution of atmospheric oxygen (O_2_) over the Phanerozoic (45). The black dashed line in (*A*) represents the threshold Fe_HR_/Fe_T_ value used to identify anoxic depositional environments (13). The red line in (*A*) is a 100 Ma running mean. The yellow lines represent LOESS smooth curves (span = 0.3). The red line in (*D*) denotes the reference seawater curve defined by (46–48). Mo_XS_ was calculated from bulk Mo concentrations using Eq.**3** and an upper crustal Mo/Al of 1.87 × 10^−6^ (g g^−1^) (49) to define the terrigenous background. The black dashed line in (*E*) indicates the K/Al of the modern terrestrial Earth’s surface (37). White and gray shaded fields (I to IV) denote the four stages of Fe cycle evolution identified in this study.

The majority of samples plotted in Fig. 1*B* show considerable variability in Fe_HR_/Fe_T_ but a relatively narrow range of Fe_T_/Al. This overall pattern is inconsistent with a pure redox control but is broadly consistent with the chemical weathering array derived from modern river suspended particles and tropical soils (Fig. 1*A*). To assess long-term (secular) changes in Earth’s surface iron cycling, we plot Fe_HR_/Fe_T_ against geologic time (Fig. 2*A*). The dataset shows considerable scatter, spanning much of the theoretical range from 0 to 1 across most intervals of Earth’s history. However, both a 100 Ma running mean and a LOESS smoothing curve reveal several notable trends, including low Neoproterozoic and early Paleozoic values, an increase in Fe_HR_/Fe_T_ from the Ordovician to the late Devonian and a decline to lower values in the late Cenozoic. The rise in mean Fe_HR_/Fe_T_ during the mid- to late Paleozoic is also reflected in an increasing baseline. In contrast, the Fe_T_/Al ratio does not display notable secular variability (see *SI Appendix*, Fig. S1*A* and *SI Appendix* for a discussion of sample populations that are consistent with the trend line of marine Fe redox cycling).

A strictly redox-centered interpretation of the rise in Fe_HR_/Fe_T_, implying a sustained intensification of marine anoxia throughout the Paleozoic, is inconsistent not only with the marine redox-cycling trend in Fig. 1, but also with multiple independent lines of evidence supporting stepwise ocean-atmosphere oxygenation during this interval. These include sulfur isotopes, trace metal systematics, thallium isotopes, and Earth system modeling (50–54). Postdepositional pyritization or alteration of poorly reactive Fe phases likely contributes to the overall scatter in Fe_HR_/Fe_T_ (Fig. 1*B*) and may also partly explain its stepwise rise during the Paleozoic (Fig. 2*A*) (41). Sulfate reduction and methanogenesis during burial are ultimately driven by organic matter degradation. Accordingly, increasing Fe_HR_/Fe_T_ through diagenetic alteration of poorly reactive Fe minerals is consistent with the general increase in organic carbon concentration from the late Ediacaran to the mid Devonian (*SI Appendix*, Fig. S1*B*). However, Fe_HR_/Fe_T_ began to rise in the Ordovician and remained elevated through the Carboniferous, an interval marked by fundamental changes in Earth surface processes. We therefore focus the following discussion on the roles of chemical weathering, physical erosion, and particle transport to the ocean in driving secular changes in sedimentary Fe speciation.

## Discussion

### Paleo-Environmental Proxy Framework.

**Fig. 3.**
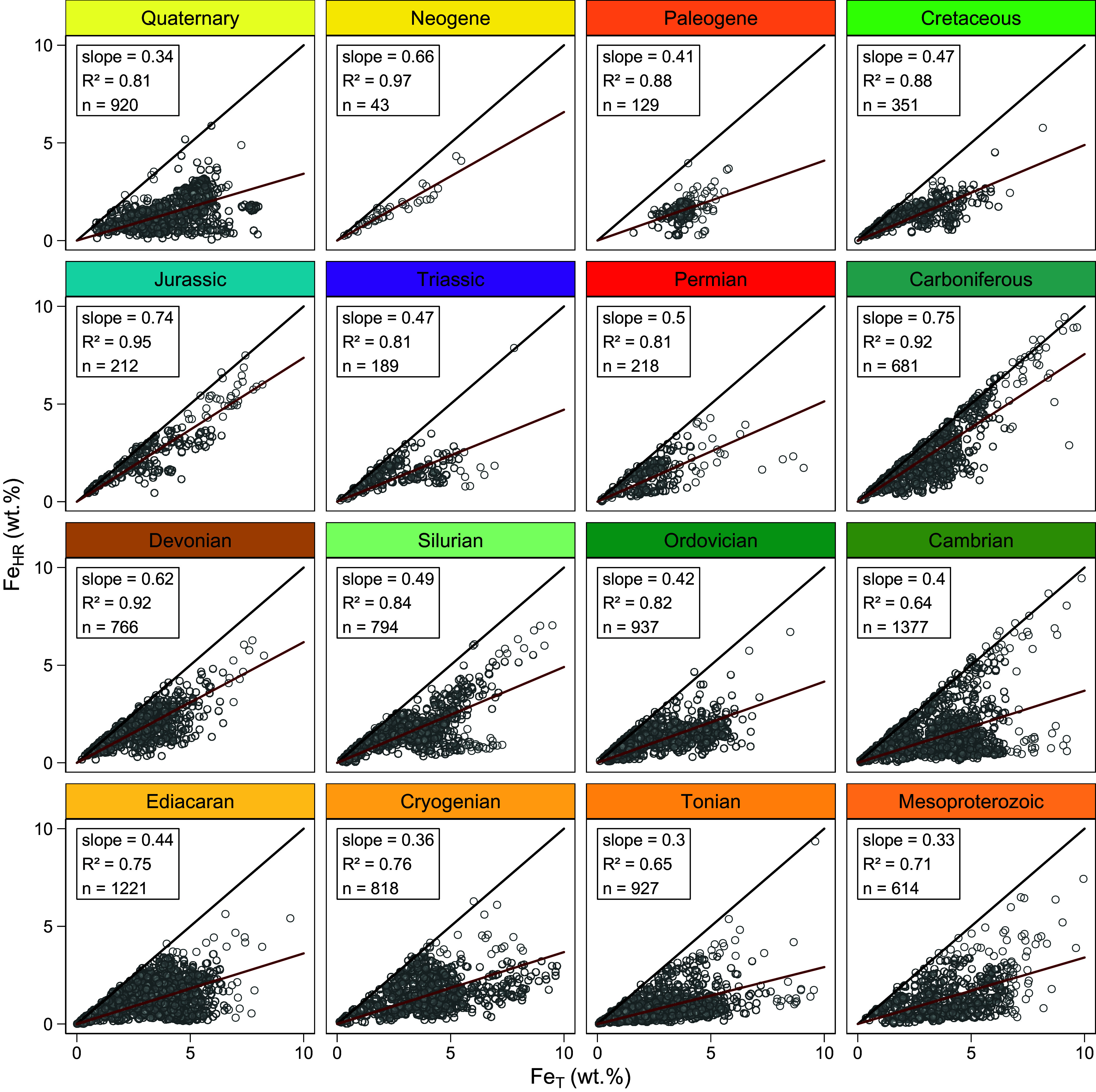
Cross-plots of highly reactive Fe vs. total Fe, subdivided by major intervals in Earth’s history. Black lines indicate Fe_HR_/Fe_T_ = 1.0. Red lines show the linear regression through all data, forced through the origin. Regression statistics are shown in the *Inset* boxes.

**Fig. 4.**
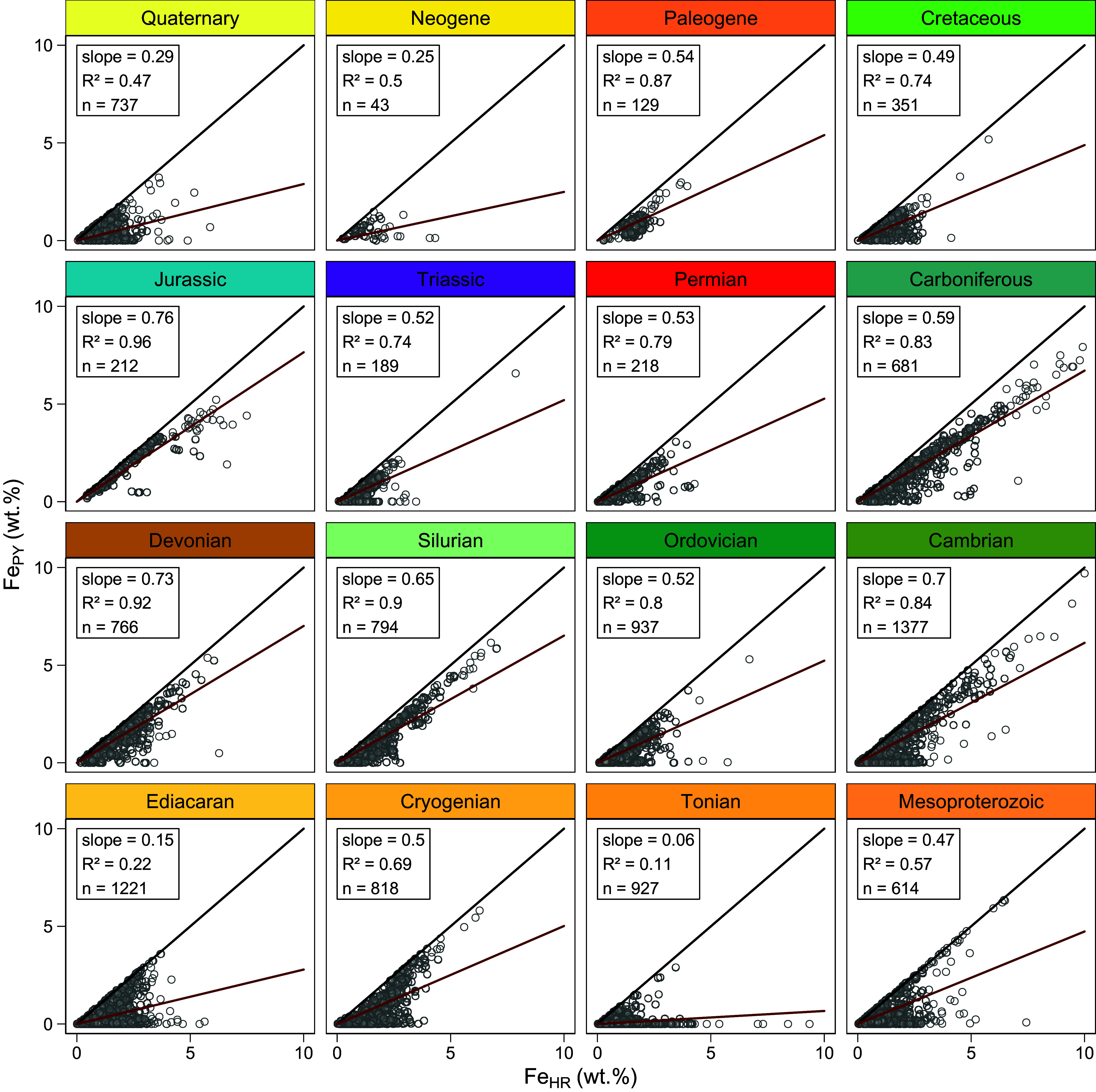
Cross-plots of pyrite Fe vs. highly reactive Fe, subdivided by major intervals in Earth’s history. Black lines indicate Fe_PY_/Fe_HR_ = 1.0. Red lines show the linear regression through all data, forced through the origin. Regression statistics are shown in the *Inset* boxes.

Building on the secular trend of Fe_HR_/Fe_T_ shown in Fig. 2*A*, we present Fe_HR_ concentrations as a function of total Fe in marine sediment samples for successive periods of Earth history since 1,200 Mya (Fig. 3). This approach allows us to assess both the magnitude of total Fe delivery to marine sediments and the extent to which this Fe was reactive toward hydrogen sulfide (6). The proportion of Fe_HR_ converted to pyrite (Fe_PY_) is shown in Fig. 2*B* and further resolved by time interval in Fig. 4. In general, a high fraction of Fe_PY_ relative to Fe_HR_ is taken to indicate that (reduced) sulfur in bottom waters or pore waters was sufficiently abundant to drive extensive pyritization of the reactive Fe supplied to the sediments.

In addition to the extent of pyritization (Figs. 2*B* and 4), independent constraints on the size of the marine sulfur reservoir are provided by secular variations in molybdenum (Mo) relative to the terrigenous background (Mo_XS_) (Fig. 2*C*). Molybdenum enters the ocean through weathering of pyrite- and organic matter-rich black shales (55) and is efficiently removed from seawater under sulfidic conditions (56). Elevated Mo_XS_ in marine sediments therefore reflects deposition under sulfidic conditions and, by inference, increased availability of both Mo and sulfur in the ocean driven by pyrite weathering on land (54, 57).

The seawater strontium isotope ratio (^87^Sr/^86^Sr) (Fig. 2*D*) reflects the relative balance between more radiogenic Sr (enriched in ^87^Sr) derived from continental silicate weathering and more unradiogenic Sr supplied by mantle-derived hydrothermal systems at mid-ocean ridges (58, 59). Elevated ^87^Sr/^86^Sr values typically indicate enhanced erosion and the release of radiogenic Sr through chemical weathering, commonly associated with continental collisions and orogenic uplift, whereas lower values reflect increased seafloor spreading and hydrothermal exchange during phases of continental rifting and breakup.

### Major Shifts in Earth Surface Iron Cycling.

**Stage I.** Mesoproterozoic through Cambrian samples exhibit the lowest mean Fe_HR_/Fe_T_ values in our dataset (0.30 to 0.44) (Fig. 3), despite widespread independent evidence for anoxic depositional conditions in contemporaneous oceans (54, 57, 60). We argue that a combination of relatively low atmospheric oxygen concentrations, absence of vegetation, and active mountain building were unfavorable for generating and preserving high proportions of reactive iron in continental margin sediments during this interval.

Intense weathering of Fe-bearing silicate minerals and the formation of highly evolved weathering products enriched in ferric Fe oxides require prolonged residence times within well-oxygenated weathering profiles. During the Mid-Proterozoic, the terrestrial Earth’s surface was largely unvegetated (61) and atmospheric oxygen concentrations remained relatively low (51). These conditions would have limited oxidative weathering of Fe-bearing silicate minerals and reduced the stabilization and retention of ferric Fe in particulate form.

A progressive rise in ^87^Sr/^86^Sr ratios from the Cryogenian through the Cambrian, linked to the break-up of the supercontinent Rodinia, the Pan-African orogeny, and the assembly of Gondwana, indicates intensified land-ocean transfer of radiogenic Sr related to enhanced continental weatherability (43, 59). Active mountain building and rapid erosion of largely unvegetated landscapes during the Pan-African orogeny would have promoted vigorous physical denudation and enhanced dissolution of readily weatherable minerals such as phosphates (62), sulfates, and sulfides (63, 64), whose dissolution rates are strongly controlled by surface exposure. At the same time, rapid erosion of relatively resistant aluminosilicate minerals likely inhibited the development of mature weathering profiles, limiting the formation and long-term preservation of Fe-oxide-rich weathering products. Amplified weathering of phosphate and sulfur minerals during the Neoproterozoic is supported by evidence for increased phosphorus availability in the ocean (65–67), as well as rising Fe_PY_/Fe_HR_ and Mo_XS_ (Fig. 2 *B* and *C*), which point to enhanced pyrite weathering on land and an expansion of the marine sulfur reservoir. However, because pyrite is itself a component of the highly reactive Fe pool, its weathering and subsequent reprecipitation as Fe oxides would not increase the net reactive Fe content of terrigenous material.

Additional evidence for relatively weak chemical weathering of aluminosilicate minerals during the Neoproterozoic and Cambrian comes from potassium (K) (Fig. 2*E*) and rubidium-to-aluminum (68) ratios in continental margin sediments. Potassium- and rubidium-rich feldspar minerals are relatively resistant to chemical weathering and therefore tend to be enriched under conditions of subdued chemical alteration (69). We observe K/Al ratios consistently well above those characteristic of the modern Earth’s surface throughout the Neoproterozoic and early Cambrian (Fig. 2*E*), which previous studies have interpreted as reflecting comparatively weak intensity of silicate weathering during these intervals (68, 69).

Simultaneously, widespread anoxia in Proterozoic oceans would have promoted the reductive dissolution of Fe oxide minerals within continental margin sediments. Analogous to processes observed in modern oxygen minimum zones (34, 70), such conditions would have facilitated the loss of sedimentary reactive Fe and its transport from continental shelves to deeper basins, ultimately contributing to relatively low Fe_HR_/Fe_T_ in continental margin sediments (Figs. 2*A* and 3).

**Stage II.** Ordovician through Devonian samples show a continuous increase in Fe_HR_/Fe_T_ from 0.40 to 0.65 (Fig. 3). A turning point in the ^87^Sr/^86^Sr record in the upper Cambrian (Fig. 2*D*) coincides with the end of the Pan-African orogeny (71) and likely reflects a shift from rapid erosion to more predominant particle storage on the continents (68). Concurrently, the emergence and evolution of land plants, beginning in the latest Cambrian and earliest Ordovician and accelerating through the Silurian (61), would have stabilized soils and river plains at the land surface (72). This stabilization greatly increased the residence time of particles in the weathering environment, allowing sufficient time for the conversion of Fe-bearing silicate minerals into reactive Fe oxide minerals. Additionally, by introducing CO_2_ and organic acids into weathering profiles, the global spread of rooted land plants from the Silurian through the Carboniferous would have accelerated silicate weathering, as evidenced by declining K/Al ratios over this interval (Fig. 2*E*) (68, 69). The concomitant rapid rise in atmospheric oxygen concentrations (Fig. 2*F*), partly driven by the proliferation of land plants, would have further intensified oxidative chemical weathering of Fe-rich aluminosilicate minerals.

**Carboniferous** samples show the highest mean Fe_HR_/Fe_T_ value of 0.75 observed in our dataset (Fig. 3). The convergence and subsequent collision of Gondwana and Laurussia during the late Devonian and Carboniferous (73) likely facilitated extensive deposition of intensely weathered, and thus Fe_HR_-rich, terrigenous sediments across widespread epicontinental seas and along continental margins, which were subsequently incorporated into the Variscan orogen within Pangaea.

**Stage III.** Permian and Mesozoic samples are comparatively sparse, limiting our ability to assess Earth surface Fe cycling during this interval. Available data suggest an initial drop in Fe_HR_/Fe_T_ across the Permian-Triassic (≤0.5), higher values in the Jurassic and again a lower mean Fe_HR_/Fe_T_ in the Cretaceous (Fig. 3). Despite these fluctuations, Fe_HR_/Fe_T_ remained above levels recorded in Mesoproterozoic through Cambrian samples (Fig. 2*A*), consistent with sustained high weathering intensity on a vegetated land surface and in the absence of major orogenic events during this interval.

**Stage IV.** Since the Paleogene, mean Fe_HR_/Fe_T_ has declined to a Quaternary value of ~0.34. Although Paleogene and Neogene samples remain comparatively sparse, the Quaternary to recent dataset is extensive, allowing us to directly compare Fe cycling on the present-day Earth surface with that of earlier intervals in Earth’s history.

Major shifts in the seawater record of ^87^Sr/^86^Sr (Fig. 2*D*) and other isotope systems during the late Cenozoic are widely attributed to increased fluxes of terrigenous sediments and solutes driven by enhanced continental weatherability associated with uplift during the Alpine orogeny (59, 74, 75). A coeval decrease in mean Fe_HR_/Fe_T_ values in marine sediments may thus indicate a global reduction in chemical weathering intensity, consistent with climatic cooling and accelerated physical erosion (76). This scenario resembles that identified in Stage I during the Pan-African orogeny. Notably, the mean Fe_HR_/Fe_T_ of our Quaternary samples is close to the mean value of 0.28 reported for modern sediments beneath oxic bottom waters reported by ref. 33, despite the inclusion of numerous anoxic basins and oxygen minimum zone settings in our dataset. This relatively low Fe_HR_/Fe_T_ in recent sediments is therefore consistent with a generally reduced extent of chemical weathering compared to much of the Phanerozoic. An exception occurs on tropical continental shelves, where substantially higher Fe_HR_/Fe_T_ values are preserved (28, 77).

### Coevolution of Surface Iron Cycling and the Earth System.

Riverine suspended particles represent by far the largest source of both total and reactive Fe to modern marine sediments (78, 79). Our analysis of Earth-surface Fe cycling over the past 1,200 Mya demonstrates that reactive Fe delivery to the oceans has varied systematically through time as a function of weathering intensity, erosion rates, and superimposed controls on these Earth system-scale processes.

The delivery of reactive Fe to the ocean is intrinsically linked to the long-term carbon cycle and the trajectory of Earth’s oxygenation through its control on pyrite formation and burial (9, 10). Moreover, reactive Fe oxides that escape pyritization can promote organic carbon burial (12). Burial of 1 mol of Fe_PY_ releases ~4 mol of O_2_, whereas burial of 1 mol of organic carbon releases 1 mol of O_2_. The relative importance of these Fe_HR_ burial pathways for oxygenation therefore depends on the extent of reactive Fe pyritization (Figs. 2*B* and 4) and the Fe:C stoichiometry of mineral-associated organic matter.

Variation in erosion rate could partially attenuate the influence of chemical weathering on Earth’s oxygenation. During intervals of rapid erosion associated with mountain building, shortened mineral residence times in weathering environments may limit the transformation of Fe-bearing silicates into Fe oxides, potentially producing an inverse relationship between the intensity of chemical weathering recorded in terrigenous particles and the total flux of terrigenous material delivered to the ocean. Nonetheless, Fe_T_, Fe_HR_, and Fe_PY_ covary through much of Earth history (Figs. 3 and 4), indicating that total iron supply, reactive iron flux, and pyrite burial have remained strongly coupled over geologic timescales. This coupling is further supported by evidence that Fe oxidation accelerates both silicate weathering and the physical fragmentation of Fe-bearing rocks, thereby enhancing the production and mobilization of reactive Fe (80).

The connection between Earth surface Fe cycling and pyrite burial was particularly pronounced during the Devonian and Carboniferous, when elevated Fe_HR_/Fe_T_ and Fe_PY_/Fe_HR_ ratios (Fig. 2), together with high R^2^ values relative to most other periods (Figs. 3 and 4), are observed. During this interval, the proliferation of land plants, amplified chemical weathering, rapid erosion associated with the Variscan orogeny, abundant seawater sulfate (81), and widespread anoxia in closing ocean basins in between Gondwana and Laurussia (73, 82, 83) created highly favorable conditions for the transfer of reactive Fe from land to ocean and enhanced pyrite burial.

We observe a striking correspondence between the mid- to late Paleozoic rise in atmospheric oxygen and the mean Fe_HR_/Fe_T_ in continental margin sediments (Fig. 2 *A* and *F*). Because Earth-surface Fe cycling is both oxygen-dependent and contributes to oxygen production through its role in pyrite and organic matter burial, this correspondence is likely to represent a positive Earth system feedback. The oxidative conversion of Fe-bearing silicate minerals to Fe oxides was accelerated by both the expansion of land plants and rising atmospheric oxygen, while oxygen accumulation was further reinforced by land-plant proliferation, enhanced chemical weathering, and pyrite burial. This cascade of interlinked processes indicates that the major shifts in Earth-surface Fe dynamics documented here were instrumental in driving Earth system evolution over hundreds of millions of years.

## Methods

### Database and Geochemical Methods.

Our analysis is largely based on the database of the SGP (39). Of the data used from the SGP database, 97% have a marine origin. Because this study focuses on Earth surface processes rather than marine Fe cycling per se, a small number of lacustrine samples were retained. Most samples belong to the lithological categories shale, mudstone or siltstone and mixed siliciclastic sedimentary rocks (93%) with minor contributions from pure carbonates (3.5%) and several other categories included in the SGP classification.

In addition to the SGP data, we incorporated a published compilation of modern marine sediments (84) and supplementary studies reporting Fe speciation data for modern marine sediments (28, 34, 85), as well as for ancient sediments from the late Quaternary (86), Pliocene (86), Paleogene (87), and Cretaceous (35) (see *SI Appendix*, Fig. S2 for sample locations). Rigorous selection criteria were employed during assembly of the dataset. The availability of Fe_HR_ (see below for details on extraction protocols), as well as total Fe and Al concentrations, was chosen as a minimum requirement for sample inclusion. Assuming a maximum permissible error of 10% for the extraction protocols, samples with Fe_HR_/Fe_T_ > 1.1 or Fe_PY_ > Fe_HR_ × 1.1 were excluded. Wherever possible, additional major element data, especially K and Mo concentrations, were also compiled. The complete data compilation, including original SGP-derived information on environmental settings, lithology, and metamorphic history, is provided in *SI Appendix*.

Iron speciation data for marine sediment, suspended particulate matter, and sediment samples were generated by applying a sequential extraction scheme (14). The most recent version of this method (15) comprises three extraction steps: i) sodium acetate to target Fe bound to carbonate minerals, ii) sodium dithionite to extract Fe oxides and hydroxides, and iii) ammonium oxalate to dissolve magnetite. The river suspended particulate matter samples (25, 26) and some of the older data in our compilation (33) were generated using a single-step sodium dithionite extraction. For these samples, the highly reactive Fe content may be underestimated compared to samples subjected to the full extraction procedure (13, 28, 88). However, because these data were used to define the threshold value for anoxia widely applied in paleo-environmental studies, we chose to retain them in our analysis. Pyrite and other reduced sulfur species were extracted using a chromous chloride distillation (89). Reduced sulfur liberated during the extraction was determined either gravimetrically or photometrically and subsequently converted to Fe_PY_ assuming a molar sulfur-to-Fe stoichiometry of 2:1.

The minerals targeted by the sequential extraction are operationally defined. It has been shown that a range of both ferrous and ferric silicate minerals, which, by definition, are not part of the highly reactive Fe pool, can partially be dissolved during the sequential extraction procedure (90–92). Because partially soluble silicate minerals are likely unevenly distributed within our data compilation, the unintended extraction of Fe from these phases may contribute to the scatter observed across most intervals of Earth’s history (Fig. 2*A*).

Previously unpublished Fe speciation data for tropical soils were generated using the same techniques described above for sediment samples. Tropical soils on granitic and basaltic bedrock were sampled at noneroding plateau positions beneath pristine primary rain forest in Uganda (0°37′N, 30°23′E) and the Democratic Republic of the Congo (2°19′S, 28°42′E), respectively. According to the World Reference Base for Soil Resources these tropical soils are classified as ferralsols. The samples were taken in 10 cm depth intervals from the uppermost 100 cm of freshly excavated soil profiles. Total Fe and Al concentrations were determined by inductively coupled plasma optical emission spectroscopy (ICP–OES) after digestion in nitric, perchloric, and hydrofluoric acids. Fe concentrations in extraction solutions were also determined by ICP–OES. The soil dataset is provided in *SI Appendix*.

### Framework for the Evaluation of Iron Redox Cycling.

Terrigenous particles delivered to the ocean carry a characteristic Fe-to-Al ratio (Fe_T,in_/Al_in_) and a defined fraction of highly reactive Fe (Fe_HR,in_/Fe_T,in_). These primary proportions may be modified after deposition through marine Fe redox cycling. Because Al remains effectively immobile during early diagenesis, whereas reactive Fe can be either added or removed via reductive dissolution and reprecipitation, changes in Fe speciation follow predictable mass–balance relationships (18).

Addition of excess reactive Fe (Fe_XS_) from sedimentary or hydrothermal sources modifies both the bulk Fe_T_/Al ratio and the reactive Fe fraction according to:

[1] $$\frac{{\mathrm{Fe}}_{T}}{\mathrm{Al}}=\frac{{\mathrm{Fe}}_{T,\mathrm{in}}+{\mathrm{Fe}}_{\mathrm{XS}}}{{\mathrm{Al}}_{\mathrm{in}}},$$

[2] $$\frac{{\mathrm{Fe}}_{\mathrm{HR}}}{{\mathrm{Fe}}_{T}}=\frac{{\mathrm{Fe}}_{\mathrm{HR},\mathrm{in}}+{\mathrm{Fe}}_{\mathrm{XS}}}{{\mathrm{Fe}}_{T,\mathrm{in}}+{\mathrm{Fe}}_{\mathrm{XS}}}.$$

If Fe is lost from the sediment through reductive dissolution and subsequent release across the sediment–water interface, Fe_XS_ becomes negative. Solving Eqs. **1** and **2** for Fe_XS_ gives

[3] $${\mathrm{Fe}}_{\mathrm{XS}}={\mathrm{Al}}_{\mathrm{in}}∙\left(\frac{{\mathrm{Fe}}_{T}}{\mathrm{Al}}\right)-{\mathrm{Fe}}_{T,\mathrm{in}},$$

[4] $${\mathrm{Fe}}_{\mathrm{XS}}=\frac{{\mathrm{Fe}}_{\mathrm{HR},\mathrm{in}}-\frac{{\mathrm{Fe}}_{\mathrm{HR}}}{{\mathrm{Fe}}_{T}}∙{\mathrm{Fe}}_{T,\mathrm{in}}}{\frac{{\mathrm{Fe}}_{\mathrm{HR}}}{{\mathrm{Fe}}_{T}}-1}.$$

Eq. **3** provides a general expression for quantifying the excess (or deficit) of any redox-sensitive element relative to its terrigenous background when normalized to Al (e.g., Mo_XS_ in Fig. 2*E*). Equating Eqs. **3** and **4** and solving for Fe_HR_/Fe_T_ yields:

[5] $$\frac{{\mathrm{Fe}}_{\mathrm{HR}}}{{\mathrm{Fe}}_{T}}=\frac{{\mathrm{Fe}}_{\mathrm{HR},\mathrm{in}}+{\mathrm{Al}}_{\mathrm{in}}∙\frac{{\mathrm{Fe}}_{T}}{\mathrm{Al}}-{\mathrm{Fe}}_{T,\mathrm{in}}}{{\mathrm{Al}}_{\mathrm{in}}∙\frac{{\mathrm{Fe}}_{T}}{\mathrm{Al}}}.$$

Eq. **5** defines the theoretical Fe-speciation trend expected from addition or removal of reactive Fe during marine redox cycling (curved line in Fig. 1). It therefore provides a quantitative framework for interpreting deviations from the terrigenous baseline and diagnosing redox-driven Fe mobilization or immobilization in marine sediments. The trend line in Fig. 1 depicting marine Fe redox cycling was calculated using Eq. **5**, with the Fe_HR_/Fe_T_ ratio of modern marine sediments underlying oxic bottom water as defined by (33) and the Fe_T_/Al of the modern terrestrial Earth’s surface (37) as the terrigenous background (Fe_T,in_ = 1.0, Fe_HR,in_ = 0.28, Al_in_ = 1.79). Adoption of an alternative terrigenous background would shift the position and curvature of the trend line. General guidelines and illustrative examples for applying Eq. **5** to evaluate redox- versus weathering-controls on Fe speciation are given in *SI Appendix*.

## Supplementary Material

Appendix 01 (PDF)

Dataset S01 (CSV)

Dataset S02 (XLSX)

## Data Availability

All study data are included in the article and/or supporting information.
