# Supplementary material for: The evolution of the Earth’s surface iron cycle
Source: Proc Natl Acad Sci U S A. 2026 Aug 5;123(32):e2608784123. doi: 10.1073/pnas.2608784123 (PMC13462457; doi:10.1073/pnas.2608784123)
Supplement: Supplementary file 1 [file pnas.2608784123.sapp.pdf]

PNAS

# Supporting Information for

## The Evolution of the Earth's Surface Iron Cycle

Florian Scholz[a,1], Sebastian Doetterl[b], Dalton S. Hardisty[c]

[a]Department of Earth System Sciences, University of Hamburg, 20146 Hamburg, Germany

[b]Department of Environmental Systems Science, ETH Zürich, 8092 Zürich, Switzerland

[c]Department of Earth and Environmental Sciences, Michigan State University, MI 48824, USA

[1]To whom correspondence may be addressed. Email: florian.scholz@uni-hamburg.de

**This PDF file includes:**

- Supporting Text
- Figures S1 to S5
- Table S1
- SI References

**Other supporting materials for this manuscript include the following:**

- Datasets S1. Iron speciation database spanning Earth's history since 1,200 Ma.
- Dataset S2. Tropical soil data.

**Supporting Text: Discussion of $Fe_T$/Al Ratios across Earth History**

Samples affected by Fe shuttling under anoxic conditions are expected to display not only elevated $Fe_{HR}/Fe_T$ but also elevated $Fe_T$/Al ratios (1, 2). In our data compilation, this condition is met for only a small fraction of samples. The mean $Fe_T$/Al of 0.57 ± 0.63 (1 SD, n = 10,197) is close to the mean $Fe_T$/Al of the modern Earth's surface (0.56) (ref. 3).

We calculated the mean $Fe_T$/Al, standard deviation, and the number of samples exceeding the global mean + SD for each period in Earth history examined in this study (Table S1). The percentage of samples exceeding this threshold ranges from 0 % (Paleogene) to 15 % (Permian) and shows no clear temporal trend across Earth history. A plot of $Fe_T$/Al against age (Figure S1A) further demonstrates that total Fe enrichment relative to Al does not follow a pronounced secular trend, in contrast to $Fe_{HR}/Fe_T$ (Fig. 2A). Moreover, the regression slope of $Fe_T$ versus Al remains similar across all investigated periods and closely matches that of the modern Earth's surface (Fig. S3). These observations suggest that the $Fe_T$/Al ratio of terrigenous sediments has remained largely constant over the time interval examined in this study.

To assess variability in Fe and Al, we also calculated the mean, SD, and coefficient of variation (CV = SD/mean) for both elements (Table S1). Across the full dataset and within most individual time intervals $CV_{Fe}$ exceeds $CV_{Al}$, consistent with greater mobility of Fe relative to Al in Earth surface environments. Overall, secular variations in Fe and Al concentrations are modest (Fig. S1C, D). A slight decline in both elements during the Cambrian may reflect reduced terrigenous sediment input following the cessation of the Pan-African orogeny. In addition, a coeval increase in total organic carbon (TOC) (Fig. S1B) suggests that dilution by enhanced biogenic sedimentation may have also contributed to the decrease in terrigenous element concentrations.

Variability in $Fe_T$ relative to Al, expressed as departures toward both higher and lower $Fe_T$/Al values relative to the modern Earth's surface, is most pronounced during the Tonian, Ediacaran, Devonian and Carboniferous (based on differences in $CV_{Fe}$ and $CV_{Al}$ in Table S1 and the data spread in Fig. S3). Some corresponding data points fall close to the trend line for marine Fe redox cycling (Fig. S4), potentially reflecting excess Fe delivery by redox-dependent Fe shuttling under conditions of sustained anoxia.

To estimate the prevalence of such samples within individual periods, we quantified the proportion of data points that exceed both the canonical anoxia threshold of $Fe_{HR}/Fe_T$ = 0.38 and the corresponding $Fe_T$/Al value of 0.65 defined by the marine Fe redox cycling trend (Table S1; red dashed boxes in Fig. S4). The highest proportion is observed during the Jurassic (57 %), followed by the Carboniferous (31 %) and Devonian (21 %). The elevated Jurassic proportion may reflect the relatively small sample size and a sampling bias toward the Toarcian Anoxic Event. The high proportion of samples influenced by Fe shuttling during the Devonian and Carboniferous is consistent with the paleogeographic configuration, intensified pyrite burial (Fig. 4), and associated oxygen release during this interval (see main article). Importantly, most of these samples plot well above the trend line, suggesting combined influences of intensified chemical weathering and redox-driven iron shuttling processes (e.g., ref. 3).

Despite these observations, the vast majority of samples across all periods (84 %) are characterized by relatively low $Fe_T/Al$ values. In contrast, $Fe_{HR}/Fe_T$ displays substantially greater variability, a pattern that more closely follows the chemical-weathering trend (Fig. S4). Collectively, these observations support the interpretation that secular variability in $Fe_{HR}/Fe_T$ is primarily driven by changes in chemical weathering and tectonically induced erosion, whereas redox-controlled processes associated with sustained anoxia exerted a secondary influence.

**Supporting Text: Assessing Redox versus Weathering Controls on Sedimentary Iron Speciation**

In Fig. S5, the Fe speciation data compiled in this study are plotted together with a series of trend lines representing marine Fe redox cycling, calculated using Equation (5) under two contrasting terrigenous input scenarios corresponding to the mean $Fe_T/Al$ of granite and basalt. These scenarios represent felsic and mafic terrigenous endmembers, respectively, and encompass the vast majority of $Fe_T/Al$ ratios observed in our compilation.

Movement along the y-axis toward higher $Fe_{HR}/Fe_T$ values at constant $Fe_T/Al$ reflects the effects of terrestrial chemical weathering or post-depositional alteration, as identified in this study. In contrast, movement toward lower $Fe_{HR}$/FeT values at constant $Fe_T/Al$ indicates a reverse-weathering trend, whereby reactive Fe is incorporated into authigenic clay minerals (e.g., ref. 4).

The full range of variability in $Fe_T/Al$ and $Fe_{HR}/Fe_T$ observed in the compiled dataset can be explained by varying combinations of terrigenous source composition (i.e., more mafic versus more felsic inputs), the intensity of terrestrial chemical weathering, redox-dependent gains or losses of Fe, and post-depositional processes such as weathering and reverse weathering. For studies targeting specific stratigraphic sections or time intervals, applying a range of $Fe_T/Al$ and $Fe_{HR}/Fe_T$ input values to Equation (5) provides a useful framework for evaluating whether observed Fe speciation trends are consistent with redox-driven Fe cycling or are more plausibly attributable to variations in sediment provenance, terrestrial chemical weathering, and/or post-depositional alteration (2). In addition, fitting a redox trend line to paleo-Fe speciation data can help constrain the local background $Fe_{HR}/Fe_T$ ratio, providing a site-specific baseline against which authigenic Fe enrichments or depletions can be evaluated (3).

**Table S1.** Statistical analysis of Fe and Al concentrations across Earth history.

| Period | n | Mean Fe (wt.%) | SD | Mean Al (wt.%) | SD | $CV_{Fe}$ | $CV_{Al}$ | mean $Fe_T Al$ | SD | n $Fe_T/Al$ > global mean + SD | (%) | n $Fe_T/Al > 0.65$ & $Fe_{HR}/Fe_T > 0.38$ | (%) |
|---|---|---|---|---|---|---|---|---|---|---|---|---|---|
| Quaternary | 836 | 4.02 | 1.56 | 6.78 | 2.13 | 0.39 | 0.31 | 0.59 | 0.15 | 4 | 0 | 86 | 9 |
| Neogene | 43 | 2.24 | 1.33 | 4.13 | 2.26 | 0.59 | 0.55 | 0.70 | 1.02 | 2 | 5 | 5 | 12 |
| Paleogene | 129 | 3.85 | 0.70 | 9.12 | 2.53 | 0.18 | 0.28 | 0.46 | 0.17 | 0 | 0 | 13 | 10 |
| Cretaceous | 351 | 2.50 | 1.60 | 5.22 | 2.78 | 0.64 | 0.53 | 0.52 | 0.29 | 13 | 4 | 50 | 14 |
| Jurassic | 212 | 3.43 | 1.90 | 5.12 | 2.86 | 0.55 | 0.56 | 0.73 | 0.27 | 8 | 4 | 121 | 57 |
| Triassic | 189 | 2.72 | 1.52 | 5.15 | 2.28 | 0.56 | 0.44 | 0.58 | 0.53 | 7 | 4 | 27 | 14 |
| Permian | 218 | 2.45 | 1.54 | 4.32 | 2.27 | 0.63 | 0.53 | 0.73 | 0.60 | 33 | 15 | 37 | 17 |
| Carboniferous | 681 | 3.51 | 2.34 | 6.14 | 2.93 | 0.67 | 0.48 | 0.74 | 1.11 | 67 | 10 | 211 | 31 |
| Devonian | 766 | 2.67 | 1.70 | 4.88 | 2.16 | 0.63 | 0.44 | 0.58 | 0.52 | 22 | 3 | 160 | 21 |
| Silurian | 794 | 3.27 | 1.71 | 6.66 | 2.65 | 0.53 | 0.40 | 0.49 | 0.21 | 8 | 1 | 85 | 11 |
| Ordovician | 937 | 2.42 | 1.57 | 5.08 | 2.79 | 0.65 | 0.55 | 0.51 | 0.32 | 29 | 3 | 100 | 11 |
| Cambrian | 1,377 | 3.60 | 2.08 | 7.27 | 3.35 | 0.58 | 0.46 | 0.60 | 1.16 | 56 | 4 | 193 | 14 |
| Ediacaran | 1,221 | 3.43 | 2.24 | 7.62 | 3.38 | 0.65 | 0.44 | 0.53 | 0.41 | 47 | 4 | 163 | 13 |
| Cryogenian | 818 | 4.10 | 1.84 | 7.31 | 2.68 | 0.45 | 0.37 | 0.64 | 0.44 | 32 | 4 | 159 | 19 |
| Tonian | 927 | 2.98 | 2.04 | 7.19 | 2.81 | 0.68 | 0.39 | 0.47 | 0.58 | 20 | 2 | 74 | 8 |
| Mesoproterozoic | 614 | 3.99 | 2.00 | 7.31 | 2.88 | 0.50 | 0.39 | 0.60 | 0.32 | 22 | 4 | 106 | 17 |
| Total | 10,197 | 3.33 | 1.98 | 6.56 | 3.02 | 0.59 | 0.46 | 0.57 | 0.63 | 370 | 4 | 1,590 | 16 |

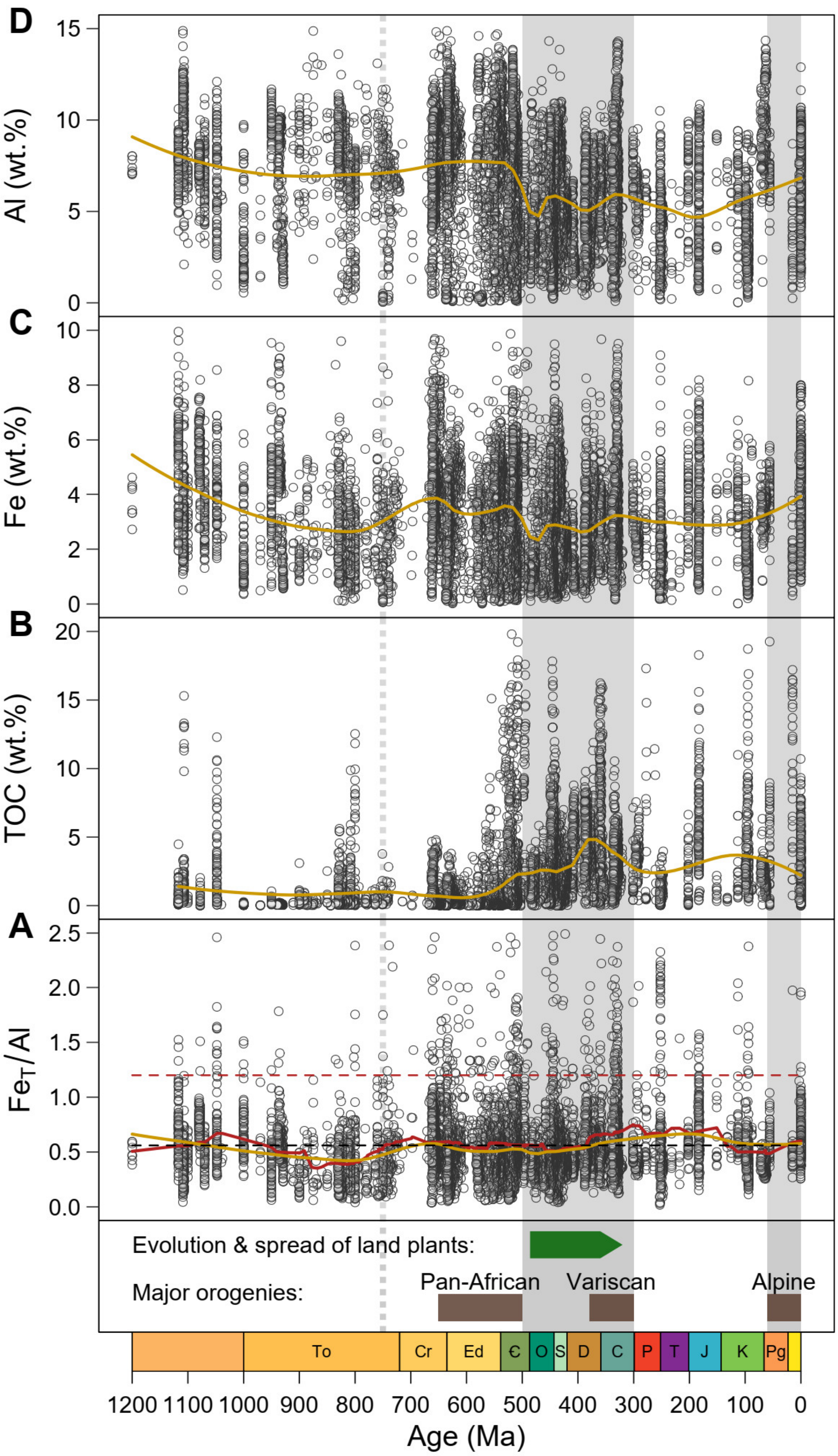


**Fig. S1.** Supplementary geochemical data plotted against age. (A) $Fe_T/Al$; (B) total organic carbon (TOC); (C) Fe; (D) Al. The red line in (A) is a 100 Ma running mean. The yellow lines represent LOESS smooth curves (span = 0.3). The black dashed line in (A) indicates the $Fe_T/Al$ of the modern terrestrial Earth's surface (5). The red dashed line indicates the mean $Fe_T/Al$ + SD.

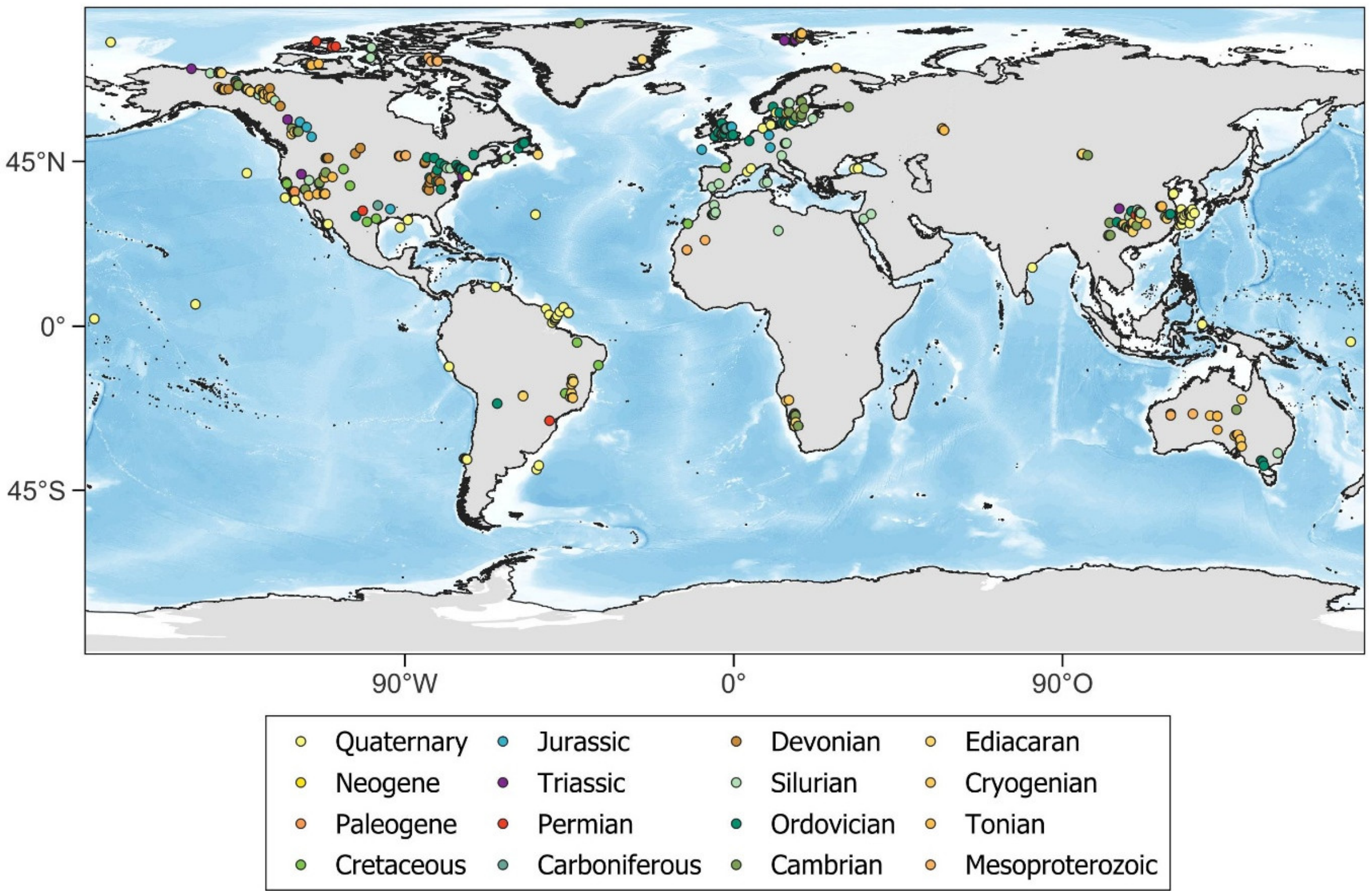


**Fig. S2.** Global map showing the geographic distribution of samples included in this study. Note that some coordinates for data drawn from the literature are approximate, and coordinates were unavailable for some samples.

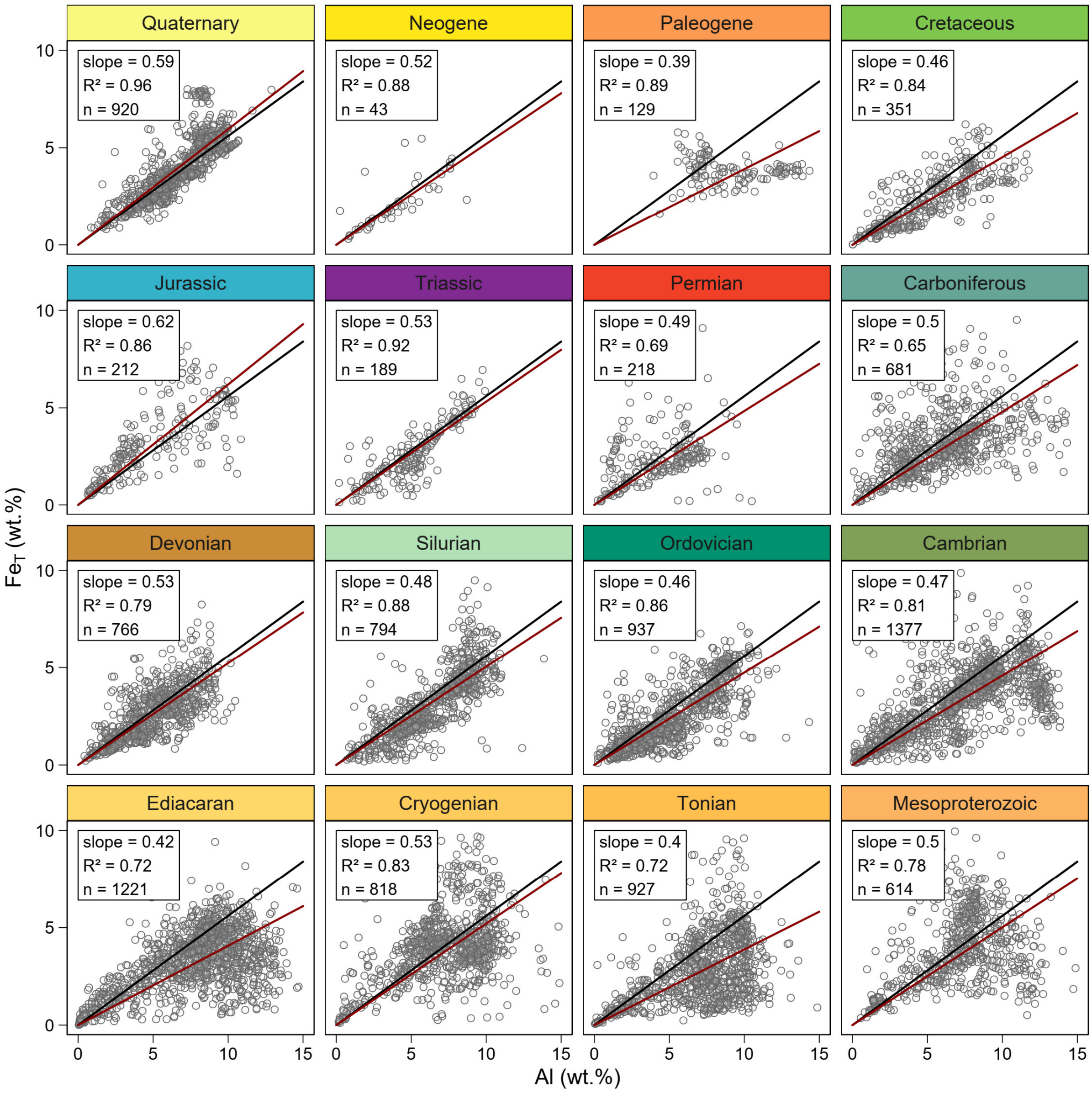


**Fig. S3.** Cross-plots of total Fe versus total Al, subdivided by major intervals in Earth's history. Black lines indicate the $Fe_T$/Al of the modern terrestrial Earth's surface (5). Red lines show the linear regression through all data, forced through the origin. Regression statistics are shown in the inset boxes.

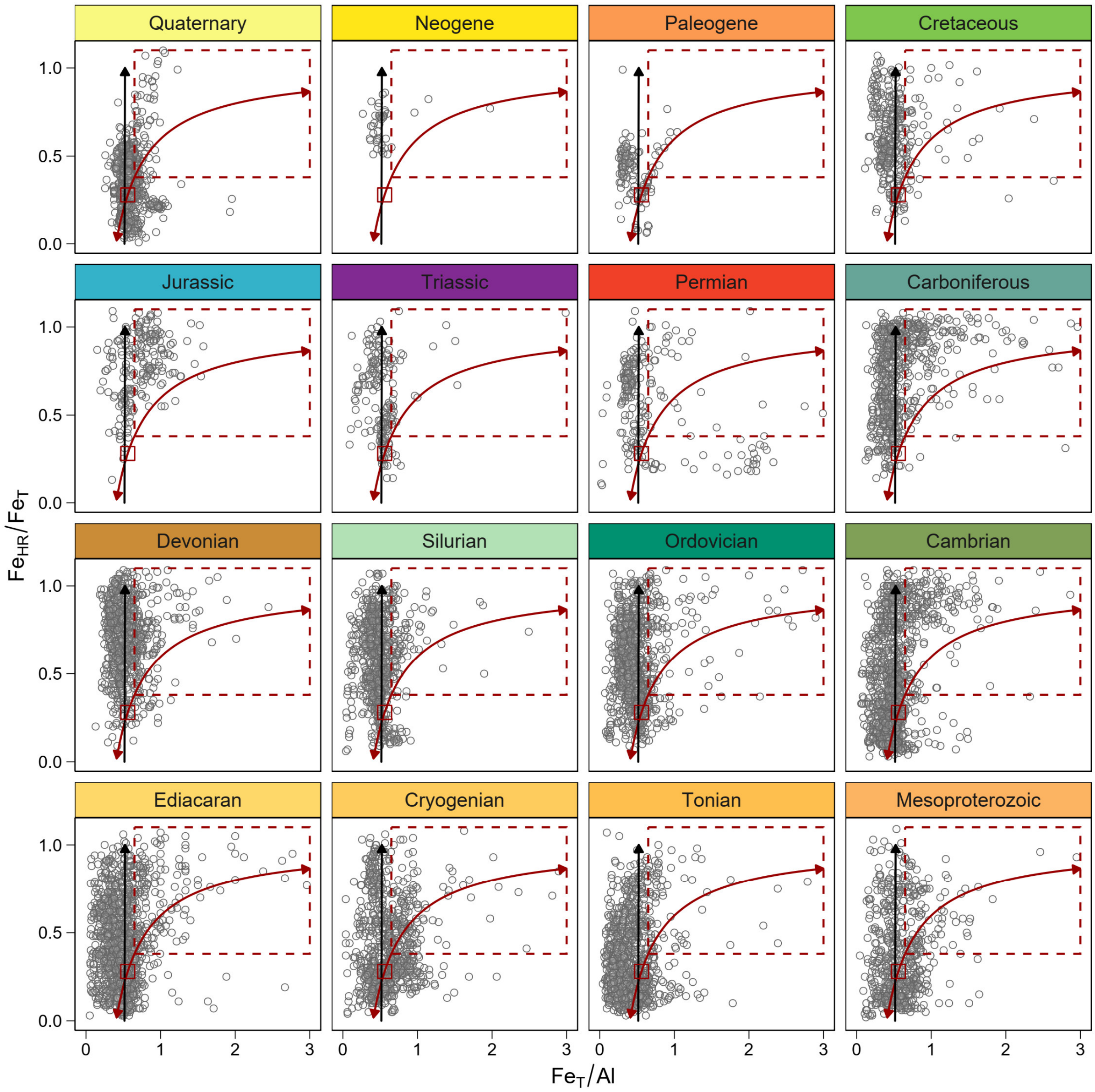


**Fig. S4.** Cross-plots of $Fe_{HR}/Fe_T$ versus $Fe_T/Al$, subdivided by major intervals in Earth's history. The red square represents the $Fe_{HR}/Fe_T$ ratio of modern marine sediments underlying oxic bottom water as defined by ref. 6, combined with the $Fe_T/Al$ of the modern terrestrial Earth's surface (5). The curved trend line, representing marine Fe redox cycling was calculated using Eq. (5), with the "oxic sediment" composition as the terrigenous baseline (see Fig. 1 in the main text). Adoption of an alternative terrigenous background would shift the position and shape of the trend line (Fig. S5). The near-vertical trend line illustrates the effect of chemical weathering. The red dashed boxes identify samples that exceed the conventional anoxia threshold of $Fe_{HR}/Fe_T$ = 0.38 and the corresponding $Fe_T/Al$ value of 0.65 defined by the marine Fe redox cycling trend.

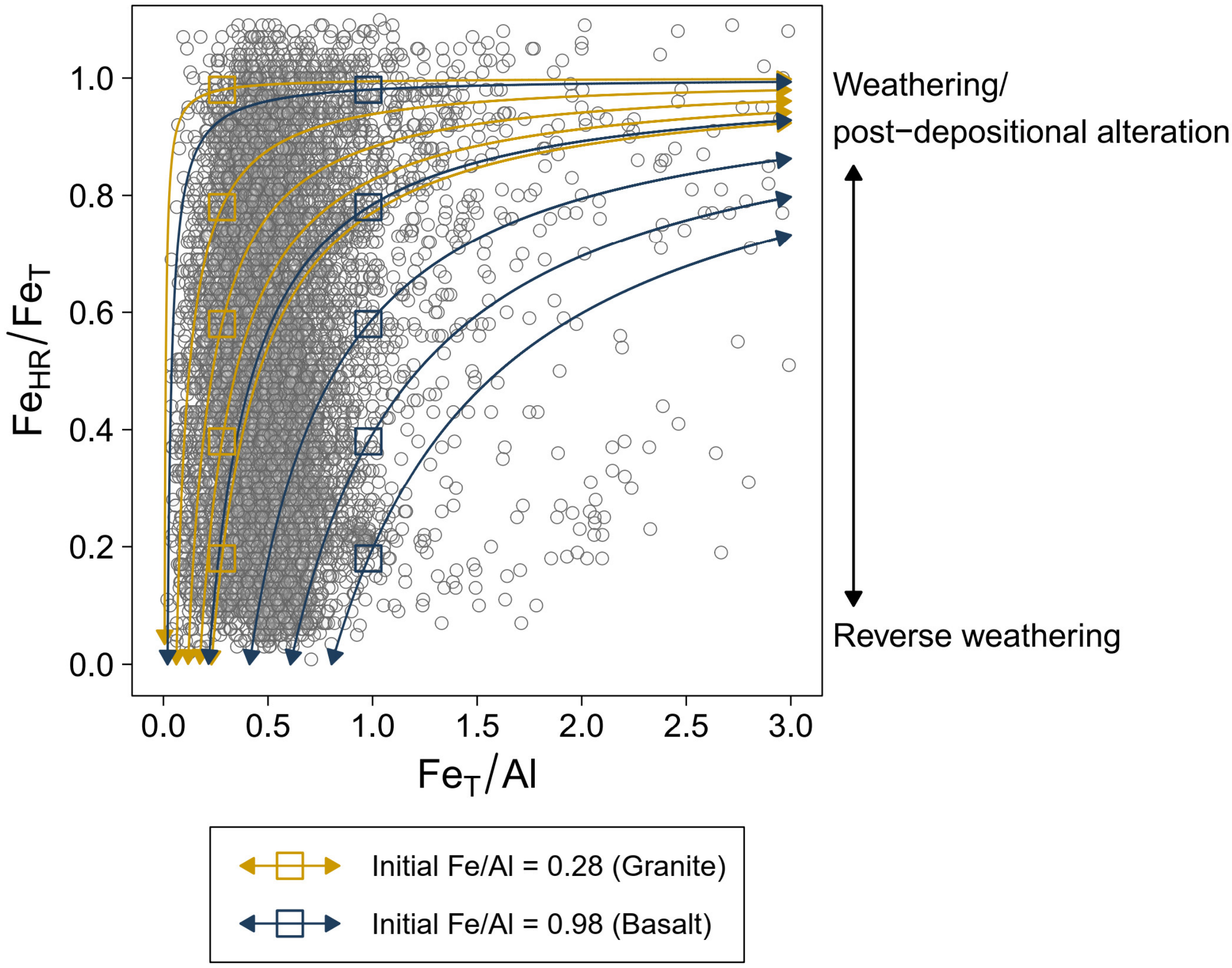


**Fig. S5.** Cross-plot of $Fe_{HR}/Fe_T$ versus $Fe_T/Al$ illustrating the combined effects of sediment provenance, terrestrial chemical weathering, marine Fe redox cycling and post-depositional processes on sedimentary Fe speciation. Trend lines of marine Fe redox cycling were calculated using Equation (5) under two contrasting terrigenous input scenarios, corresponding to the mean $Fe_T/Al$ ratios of granite and basalt (5), and a variable extent of terrestrial chemical weathering (expressed as $Fe_{HR}/Fe_T$ variability along the y-axis). Upward- and rightward-directed arrows indicate reactive Fe enrichment relative to the terrigenous input through Fe shuttling, whereas downward-directed arrows indicate reactive Fe loss associated with sedimentary Fe release. The effects of post-depositional alteration and reverse weathering are illustrated by the vertical arrow on the right.

**SI References**


1. T. W. Lyons, S. Severmann, A critical look at iron paleoredox proxies: New insights from modern euxinic marine basins. Geochimica et Cosmochimica Acta 70, 5698–5722 (2006).
2. F. Scholz, Identifying oxygen minimum zone-type biogeochemical cycling in Earth history using inorganic geochemical proxies. Earth-Science Reviews 184, 29–45 (2018).
3. F. Scholz et al., Oxygen minimum zone-type biogeochemical cycling in the Cenomanian-Turonian Proto-North Atlantic across Oceanic Anoxic Event 2. Earth Planet. Sci. Lett. 517, 50–60 (2019).
4. P. Vosteen et al., Iron redox shuttling and uptake by silicate minerals on the Namibian mud belt. Geochimica et Cosmochimica Acta 386, 1–17 (2024).
5. J. Hartmann, H. H. Dürr, N. Moosdorf, M. Meybeck, S. Kempe, The geochemical composition of the terrestrial surface (without soils) and comparison with the upper continental crust. International Journal of Earth Sciences 101, 365–376 (2012).
6. R. Raiswell, D. E. Canfield, Sources of iron for pyrite formation in marine sediments. American Journal of Science 298, 219–245 (1998).
